# Anesthetic management of cesarean section in cases of placenta accreta, with versus without abdominal aortic balloon occlusion: study protocol for a randomized controlled trial

**DOI:** 10.1186/s13063-017-1977-5

**Published:** 2017-05-26

**Authors:** Qinjun Chu, Dan Shen, Long He, Hongwei Wang, Xianlan Zhao, Zhimin Chen, Yanli Wang, Wei Zhang

**Affiliations:** 1 Department of Anesthesiology, The First Affiliated Hospital of Zhengzhou University, Jian She Dong Lu, No 1, Zhengzhou, 450052 Henan Province China; 2 Department of Obstetrics, The First Affiliated Hospital of Zhengzhou University, Jian She Dong Lu, No 1, Zhengzhou, 450052 Henan Province China; 3 Department of Interventional Radiology, The First Affiliated Hospital of Zhengzhou University, Jian She Dong Lu, No 1, Zhengzhou, 450052 Henan Province China

**Keywords:** Placenta accreta, Cesarean section, Massive hemorrhage, Abdominal aortic balloon occlusion, Randomized controlled trial

## Abstract

**Background:**

Placenta accreta (PA), a severe complication during delivery, is closely linked with massive hemorrhage which could endanger the lives of both mother and baby. Moreover, the incidence of PA has increased dramatically with the increasing rate of cesarean deliveries in the past few decades. Therefore, studies evaluating the effects of different perioperative managements based on different modalities in the treatment of PA are necessary. Among the numerous treatment measures, prophylactic abdominal aortic balloon occlusion (AABO) in combination with cesarean section for PA seems to be more advantageous than others. However, up to now, all studies on AABO were almost retrospective. Current evidence is insufficient to recommend for or against routinely using the AABO technology for control intraoperative hemorrhage in patients with PA. Thus, we hope to carry out a prospective, randomized controlled trial (RCT) study to confirm the effectiveness of the AABO technology in patients with PA.

**Methods/design:**

This trial is an investigator-initiated, prospective RCT that will test the superiority of AABO in combination with cesarean section compared to the traditional hysterectomy following cesarean section for parturients with PA. A total of 170 parturients with PA undergoing cesarean section will be randomized to receive either AABO in combination with cesarean section or the traditional hysterectomy following cesarean section. The primary outcome is estimated blood loss. The most important secondary outcome is the occurrence of cesarean hysterectomy during delivery; others include blood transfusion volume, operating time, neonate’s Apgar scores (collected at 1, 5 and 10 min), length of stay in intensive care unit, total hospital stay, and balloon occlusion-relative data.

**Discussion:**

This prospective trial will test the superiority of AABO in combination with cesarean section compared to the traditional hysterectomy following cesarean section for parturients with PA. It may provide strong evidence about the benefits and risks of AABO in combination with cesarean section for parturients with PA.

**Trial registration:**

Chinese Clinical Trial Registry, ChiCTR-INR-16008842. Registered on 14 July 2016.

**Electronic supplementary material:**

The online version of this article (doi:10.1186/s13063-017-1977-5) contains supplementary material, which is available to authorized users.

## Background

Placenta accreta (PA), a kind of morbidly adherent placenta, involves an absence of decidua basalis; the placenta always adheres to the underlying myometrium [1]. This morbidly obstetric condition is a severe complication during delivery. It is closely linked with massive hemorrhage, which could endanger the lives of both mother and baby [2, 3]. In patients with PA, median intraoperative blood loss is about 2000 ml during delivery, and in 10% of the cases it has been more than 10,000 ml in some institutions [4, 5]. Multiple pathophysiological conditions are involved in massive hemorrhage from patients with PA during cesarean section, including pelvic viscera injury to the bladder, ureter, bowel, or other organs during the surgery. When massive hemorrhage cannot be effectively controlled, multisystem organ failure (MOF) and disseminated intravascular coagulation (DIC) will follow [6]. Therefore, placenta accreta poses a serious threat to the safety of the mother.

However, in recent years, the incidence of PA has risen with the increasing rate of cesarean deliveries [7]. According to a World Health Organization (WHO) report, China has become the country with the highest rate of cesarean sections, up to 68%. China will face a big challenge with its two-child family policy initiation. In America, the cesarean delivery rate will be 56.2% if the cesarean rate continues to grow as it has in the past, and there will be 6236 patients with placenta previa, 4504 patients with placenta accreta, and 130 maternal deaths annually [8]. Therefore, studies evaluating the effects of different perioperative management approaches based on different modalities in the treatment of placenta accreta are necessary.

In the traditional method, hysterectomy following cesarean section is usually indicated as the treatment option in patients with morbidly adherent placenta. This treatment reduces maternal morbidity, but it results in infertility [9]. To minimize intraoperative hemorrhage and facilitate surgery, some novel treatment options have been described, including proximal ligation of the internal iliac artery or uterine artery and vascular balloon catheters placed in the iliac artery, uterine artery, or even the abdominal aorta [10]. Among the numerous treatment measures, prophylactic balloon occlusion of the abdominal aorta seems to be more advantageous than others [11, 12]. Recently, a large-sample retrospective study from our single center also shows that prophylactic abdominal aortic balloon occlusion (AABO) has a promising future [13, 14]. These retrospective studies strongly suggest that we need an adequately powered prospective study to demonstrate the benefits and risks. Thus, the primary aim of the study is to investigate the benefits and risks of AABO in combination with cesarean section for PA.

## Methods/design

### Trial design

**Fig. 1:**
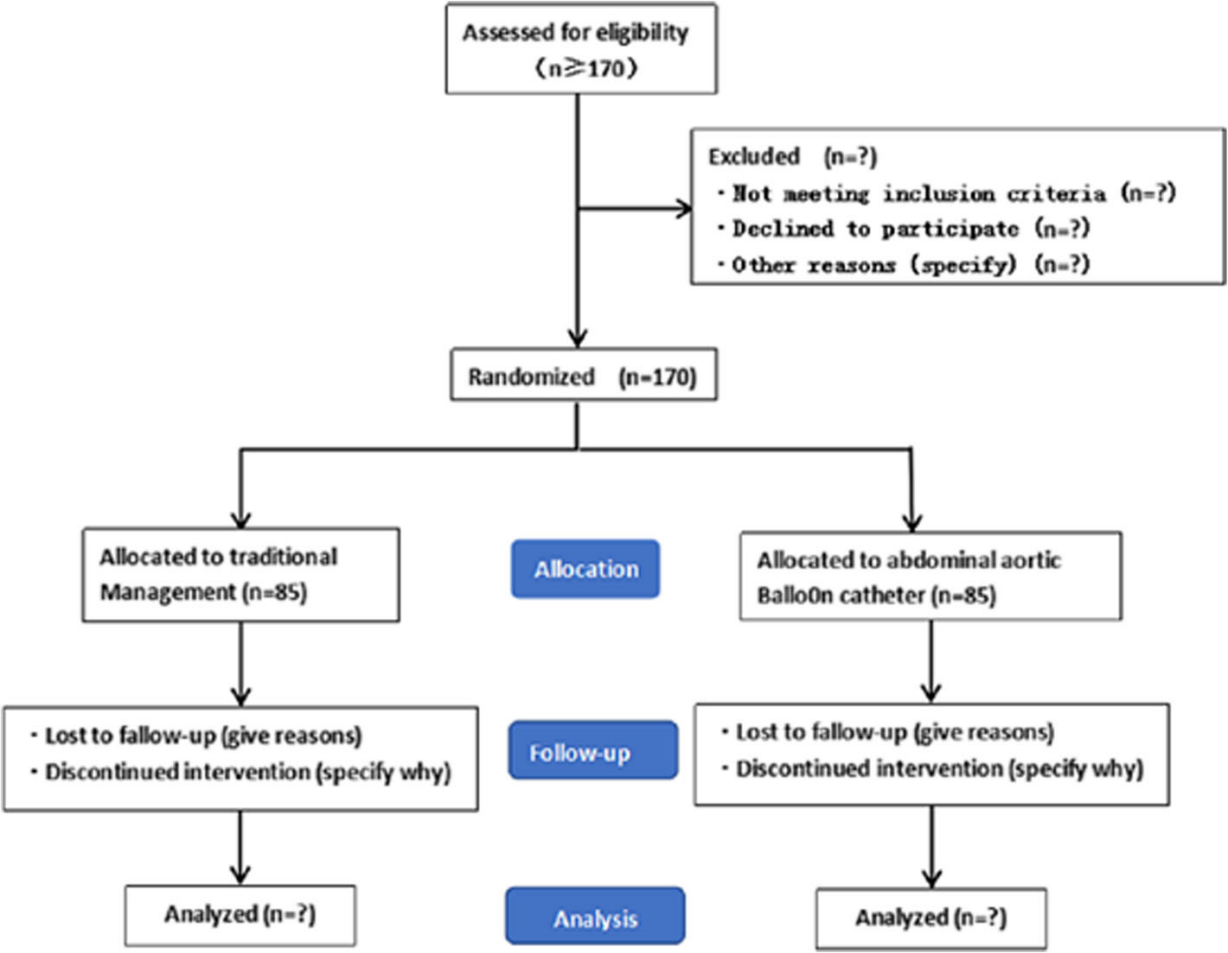
Flow diagram of the study

This protocol was developed in accordance with the Standard Protocol Items: Recommendations for Interventional Trials (SPIRIT) Statement. For the SPIRIT checklist see Additional file 1, and for the SPIRIT figure see Additional file 2. This trial is an investigator-initiated, prospective, randomized controlled trial (RCT) that will test the superiority of AABO in combination with cesarean section compared to the traditional hysterectomy following cesarean section for parturients with PA. A brief flow diagram of the study is shown in Fig. 1.

### Recruitment

From June 2016 to December 2018, a total of 170 patients diagnosed with PA, based on the obstetrician’s knowledge and experience and on an ultrasound or magnetic resonance imaging (MRI) examination [15, 16], will be enrolled in this study at the First Affiliated Hospital of Zhengzhou University.

### Enrollment criteria

All the subjects must meet the following inclusion criteria:

1. Patient diagnosed with PA based on obstetrician’s knowledge and experience and on ultrasonic or MRI examination
2. Undergoing elective or emergency cesarean section

The exclusion criteria are as follows:

1. Patient’s or relative’s refusal to participate
2. Uncontrolled sepsis or infection of femoral artery puncture site in inguinal region
3. Severe peripheral vascular disease
4. Aortic diseases including, e.g., aortoarteritis, aortic aneurysm, dissection of aorta, and aortic regurgitation, etc.
5. Severe cardiac disease

### Randomization, allocation concealment, and blinding

Subjects who meet the eligibility criteria will be randomly assigned to either the traditional treatment group or the interventional treatment group. Group assignments are generated using a computer algorithm (Winpepi version 11) that allocates patients in equal numbers to both groups. The randomization list will be sealed in sequentially numbered opaque envelopes, which will be stored in a double-locked cabinet. Randomization is implemented by a research assistant who is not involved in recruitment. After random assignment, the envelopes will again be stored separately in a double-locked cabinet. Allocation concealment will not be broken until the trial is complete.

### Study organization

The study will be supervised for data collection, security, and storage by the Department of Anesthesiology, the First Affiliated Hospital of Zhengzhou University, and Zhao XL and Zhang W will be in charge of data quality control. The institutional ethics committee from our hospital will be involved in the whole process of the trial.

### Trial interventions

All patients will receive the standard multidisciplinary team service (including the senior obstetrician, gynecologist, anesthesiologist, neonatology teams, urologist, imaging doctor, and adequate number of blood units in the operating room). The antepartum diagnosis of PA is made clinically, based on the pelvic ultrasound or MRI. Pregnant woman with PA diagnosed by ultrasound or MRI will be recruited in this study. In both groups, pregnant woman will undergo the standard protocol, as described previously [14]. Considering the risk of massive bleeding complicated by profound hypotension and coagulopathy, we choose general anesthesia for all patients with PA [17, 18]. Endotracheal intubation is performed with the use of a rapid sequence induction (RSI) technique [19, 20]. We monitor invasive arterial blood pressure (ABP), electrocardiogram (ECG), SPO_2_, PetCO_2_, temperature (T), arterial blood gas (ABG), and thrombelastograph (TEG) of the patients during the operation. After fetal delivery and umbilical cord clamping, according to the PA location and depth, patients are given local excision of the uterine wall, placenta evacuation, partial cystectomy, and bladder repair. 20 U of oxytocin and 250 μg of tromethamine are injected in the myometrium.

In the traditional group, patients are given a cesarean section without AABO. In this group, conservative treatments for PA, including oversewing of the placental bed, a uterine tamponade, and bilateral uterine artery, ligation are used. Hysterectomy is performed when massive hemorrhage cannot be controlled.

**Fig. 2:**
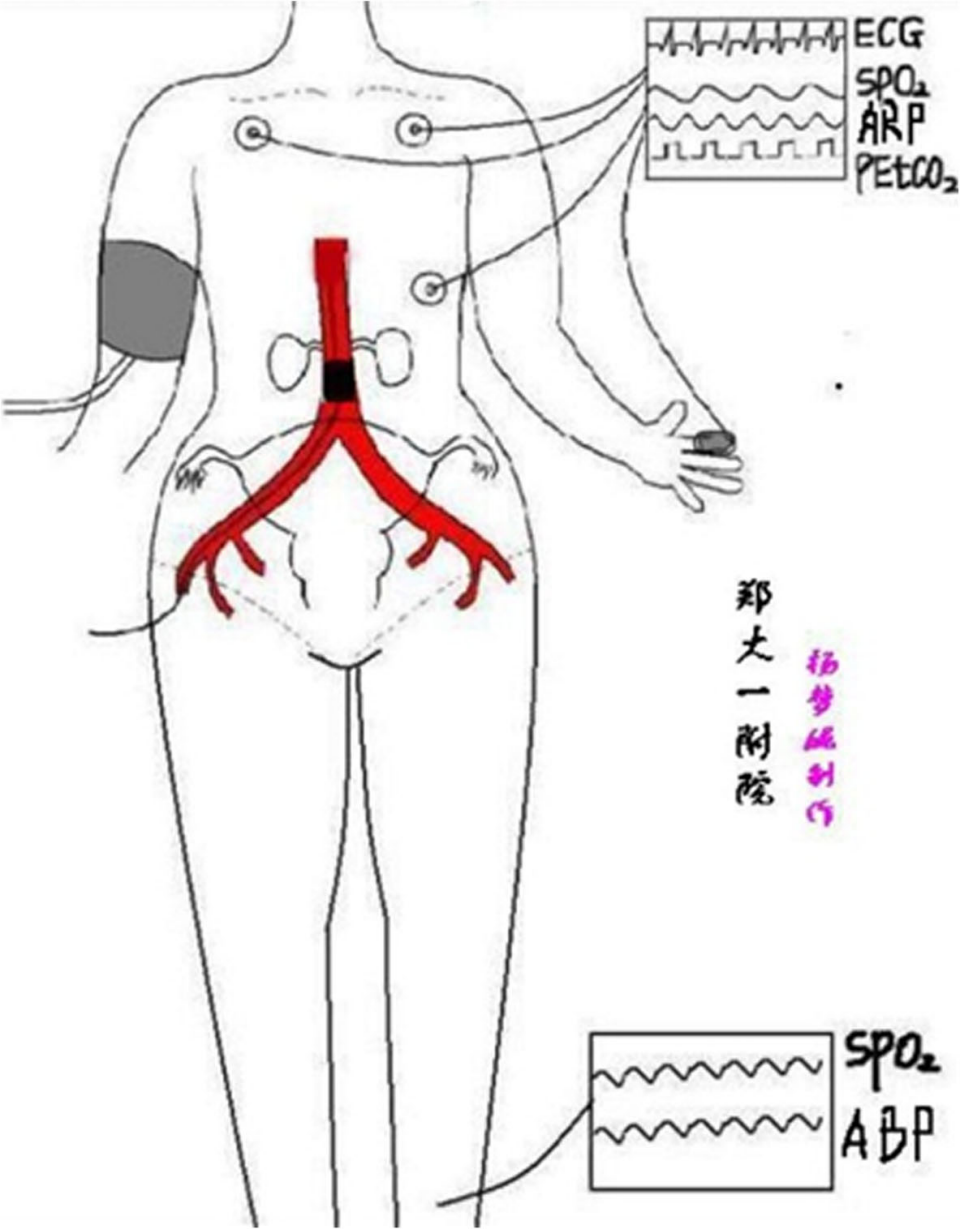
Sketch of abdominal aortic balloon position and related physiological parameter monitoring

In the interventional group, the cesarean section and all endovascular procedures will be performed in a hybrid operation room equipped with a digital subtraction angiography (DSA) machine (Allura Xper FD20, Philips, Best, the Netherlands). Interventional radiologists will select the proper diameter of the balloon, which is measured by MRI, and insert a 5 F pigtail catheter (Cook, Bloomington, IN, USA) into the abdominal aorta at the level of T12 with an 8-F sheath (Cook) from the right femoral artery at the groin, with the patient under local anesthesia. Next, 5 ml iodixanol (Visipaque-320, Nycomed, Oslo, Norway) will be injected to locate the origin of the renal arteries. An 8-F, 40 × 14 mm, 40 × 16 mm, or 40 × 18 mm balloon catheter (Bard Peripheral Vascular, Tempe, AZ, USA) will be inserted into the infrarenal abdominal aorta and fixed carefully. Each patient will have peripheral oxygen saturation placed on the great toes of the left foot to allow the interventional radiologist to determine when balloon catheter occlusion of the aorta has occurred during the endovascular procedures. Indirectly confirmed balloon block effective indicators are as follows: the digit blood oxygen is reduced to zero, the blood oxygen curve is at a flat state, and the bipedal arterial blood pressure drops to zero [13, 21]. A sketch drawing of the abdominal aortic balloon position and related monitoring of physiological parameters during the operation is shown in Fig. 2.

Temporary aortic balloon occlusion will be implemented by using 10–16 ml of saline solution immediately after fetal delivery and umbilical cord clamping. The balloons are inflated for 12–15 min, and the inflations are alternated with deflations of 1–2 min. As reported, it is safe to block the pelvic organs and lower limbs for 30 min [22]. After the operation, a pelvic angiography is performed again. If there is active bleeding, uterine artery embolization (UAE) is supplemented. The fluoroscopy time is recorded in all cases. When the operation is completed, the catheter is pulled out and compression bandaging of the femoral artery puncture sites is performed. The lower limbs of the patients are massaged after the operation. Low-molecular-weight heparin is given to the patients after 24 h to prevent vein thrombosis of the lower limbs.

### Data collection

#### Primary outcome

The primary outcome is estimated blood loss (EBL). EBL is measured with reference to the collected blood in the suction bottle in the operating room and to the weight of the surgical swabs, excluding the volume of amniotic fluid.

#### Secondary outcomes

The following are the secondary outcomes of interest in the study:

1. Occurrence of cesarean hysterectomy (CH) during delivery
2. Blood transfusion volume
3. Neonate outcome: 1, 5 and 10 min Apgar scores
4. Operating time from the time of skin incision to abdominal closure
5. Balloon occlusion-relative data: balloon occlusion time, fetal radiation dose
6. Balloon occlusion-relative complications: vascular aneurysm, dissection or rupture, arterial embolism
7. Length of stay in intensive care unit (ICU)
8. Total hospital stay

### Patient withdrawal

A participant or a patient’s relative who no longer agrees to participate in the study can withdraw at any time without need of further explanation. Patients who are withdrawn from the study protocol will not be followed up, and their data will not be analyzed. It is essential for the trial to collect as much data from each participant as possible.

### Sample size

The sample size was calculated to compare two proportions with two-sample noninferiority or superiority. According to Muñoz et al., median blood loss was 2000 ml in parturients with PA during delivery [23]. The study of Panici et al. showed that median blood loss was 950 ml in parturients with AABO at cesarean section [24]. Considering a difference in volume of bleeding of 1000 ml with a standard deviation of 355 ml [13] in two treatment groups, a difference of 800 ml between two treatment groups is considered to be clinically important. Assuming the difference between two groups at a 2.5% significance level and a power of 0.90, 67 patients in each group are required for a comparison within the groups. Considering an estimated 20% dropout rate, 85 patients in each group for a total of 170 patients will be included in this study.

### Statistics

An intention-to-treat analysis will be performed to compare all primary and secondary outcomes in the future study. Continuous variables will be described as means ± standard deviation (SD). Categorical variables will be described as percentages. Demographic data, blood transfusion volume, operating time, length of stay in the ICU, total hospital stay, and EBL will be analyzed by Student’s *t* test to compare the two methods. The occurrence of CH during delivery will be compared by the chi-square test. The neonate Apgar scores will be analyzed using the Mann-Whitney *U* test.

The statistical analyses will be carried out with SPSS software (SPSS, Inc., Chicago, IL, USA). The statistical significance will be considered with a two-tailed *P* value <0.05 and a one-tailed *P* value <0.025.

### Data processing and security monitoring

All data will be collected in accordance with the approved agreement and recorded separately. The Information Board (data monitoring committee, DMC) is composed of two senior professors, an epidemiologist, a biostatistics expert, and an ethical expert without any conflict of interest. Weekly meetings and public meetings are held to ensure data availability and scientific integrity while protecting patient safety. The main goals are to ensure the safety and interests of the subjects, the integrity and credibility of the study, and timely and accurate feedback to the clinical research related to the field. All treatment-related adverse events will be observed and reported by participants at each visit. In the event of serious adverse reactions, detailed reports will be drafted assessed on the basis of the protocol. Data and safety monitoring will be conducted regularly during the study period.

## Discussion

So far, massive hemorrhage caused by PA during delivery is still a big challenge for obstetricians. In patients with PA, if the placenta does not completely separate from the uterus during delivery, massive obstetric hemorrhage will follow, leading to DIC and to a vicious circle of bleeding. When conservative treatments fail, traditional measures for massive obstetric hemorrhage include uterine artery ligation, internal iliac artery ligation, or even emergent hysterectomy. In the last 30–40 years, a new vascular interventional technique for treatment of obstetric hemorrhage has emerged. Pelvic arterial embolization and temporary occlusion of internal iliac arteries seem to be safe and effective for massive obstetric hemorrhage [25]. However, studies have yielded conflicting results. Some studies showed these treatments could reduce blood loss, others showed no benefits, and some even showed significant complications [26, 27].

The internal iliac artery is the main blood supply to the pelvic cavity. The uterine artery usually arises from the anterior division of the internal iliac artery, which is the main supply to the uterus. However, there are several other vascular territories that provide a rich collateral supply to the uterus, such as the ovarian artery, which arises from the abdominal aorta below the renal artery [28–30]. So, theoretically, the abdominal aorta should be an ideal alternative site proposed for temporary occlusion, which may greatly diminish the collateral supply. Intra-aortic balloon occlusion (IABO) is not a new vascular interventional technique for controlling massive hemorrhage. An intraluminal aortic occlusion technique for controlling massive intra-abdominal hemorrhage was first reported by Edwards et al. in 1953 [31]. The endovascular balloon occlusion technique has been widely used in various types of major bleeding since Edwards’ report. The endovascular balloon occlusion technique has been successfully used in, e.g., trauma, aneurysm, artery dissection, and tumors, etc. [32–34]. For different regions of the aortic blood supply, Stannard et al. described in detail how different types of aortal occlusion, from the chest, to the abdominal cavity, to the pelvic cavity, can be chosen to reduce bleeding [35]. Paull et al. first introduced the abdominal aortic balloon occlusion (AABO) technology into clinical practice in the obstetrics field to control intraoperative hemorrhage [21]. Up to now, less than ten studies, with small sample sizes, using the AABO technology have been reported [36–40]. Although most of them showed positive results, all these studies were almost retrospective studies. Current evidence is insufficient to recommend for or against routinely using the AABO technology to control intraoperative hemorrhage in patients with PA. Therefore, we hope to carry out this prospective RCT study to confirm the effectiveness of the AABO technology in patients with PA.

### Trial status

The study is not yet recruiting as of the date of publication.

## Additional files

Additional file 1: SPIRIT checklist. (DOC 118 kb)

Additional file 2: Schedule of enrollments, interventions, and assessments. (DOC 63 kb)

## Abbreviations

AABO: Abdominal aortic balloon occlusion
ABG: Arterial blood gas
ABP: Arterial blood pressure
CH: Cesarean hysterectomy
DIC: Disseminated intravascular coagulation
DSA: Digital subtraction angiography
EBL: Estimated blood loss
ECG: Electrocardiogram
IABO: Intra-aortic balloon occlusion
ICU: Intensive care unit
MOF: Multisystem organ failure
MRI: Magnetic resonance imaging
PA: Placenta accreta
RCT: Randomized controlled trial
RSI: Rapid sequence induction
SD: Standard deviation
TEG: Thrombelastograph
UAE: Uterine artery embolization
WHO: World Health Organization
