# Supplementary material for: Anesthetic management of cesarean section in cases of placenta accreta, with versus without abdominal aortic balloon occlusion: study protocol for a randomized controlled trial
Source: Trials. 2017 May 26;18:240. doi: 10.1186/s13063-017-1977-5 (PMC5446702; doi:10.1186/s13063-017-1977-5)
Supplement: Supplementary file 2 — Schedule of enrollments, interventions, and assessments. (DOC 63 kb) [file 13063_2017_1977_MOESM2_ESM.doc]

Figure. Example template of recommended content for the schedule of enrolment, interventions, and assessments.*

|  | **STUDY PERIOD** | | | | | | | | |
| --- | --- | --- | --- | --- | --- | --- | --- | --- | --- |
|  | **Enrol-ment** | **Alloca-tion** | **Post-allocation** | | | | | | **Close-out** |
| **TIME-POINT**** | ***1 day before***  ***operation*** | ***1 hour before***  ***operation*** | ***Place-ment of balloon catheter*** | ***Gene-ral anes-thesia*** | ***Deli-very of baby*** | ***Pla-cental abrup-tion*** | ***Suture***  ***of the***  ***uterus*** | ***End of***  ***the surgery*** | ***Leave the operating room*** |
| **ENROL-MENT:** |  |  |  |  |  |  |  |  |  |
| **Eligibility screen** | **The inclusion criteria:**  1. Patients diagnosis as placenta accreta based on obstetrician’s knowledge, experience, and ultrasonic or MRI examination  2.Undergo-ing elective or emergency cesarean section  **The exclusion criteria:**  1.Patient’s or relative’s refusal to participate  2.Uncontrolled sepsis or infection of femoral artery puncture site in inguinal region  3.Severe peripheral vascular disease  4.Aortic diseases including aorto-arteritis, aortic aneurysm, or dissection of aorta and aortic regurgitation et al  5.Severe cardiac disease |  |  |  |  |  |  |  |  |
| **Informed consent** | All the patients will be informed of the severity of their condition, treatment options, and related risks. Written informed consent will be obtained from all the participants in the study. |  |  |  |  |  |  |  |  |
| ***[List other procedures]*** | Operation and anesthesia approval |  |  |  |  |  |  |  |  |
| **Allocation** |  | Groups assignments are generated using a computer algorithm (WinPepi version 11) that allocated patients in equal numbers to both groups. The randomisation list will be sealed in sequentially numbered opaque envelopes, where they will be stored in a double-locked cabinet. Randomization is implemented by research assistant who is not involved in recruitment. |  |  |  |  |  |  |  |
| **INTERVENTIONS:** |  |  |  |  |  |  |  |  |  |
| ***[Intervention A]*** |  |  | A balloon catheter will be inserted into the infrarenal abdomen-al aorta and be fixed carefully. | Each patient will have peripheral oxygen saturation placed on the great toes of the left foot | Tempo-rary aortic balloon occlu-sion will imple-ment by using 10–16 ml of saline solution imme-diately. | 1.The balloons are inflated for 12–15 min and the inflations are alternated with deflations of 1–2 min.  2.conser-vative treatments are used. | Hysterec-tomy is performed when massive hemorrhage cannot be controlled. | the catheter will be pulled out and compression bandaging of femoral artery puncture sites will be performed. | Low-molecular-weight heparin will be applied to the patients after 24 h |
| ***[List other study groups]*** |  |  |  |  |  | Conser-vative treatments are used. | Hysterec-tomy is performed when massive hemorrhage cannot be controlled. |  |  |
| **ASSESSMENTS:** |  |  |  |  |  |  |  |  |  |
| ***[List baseline variables]*** | Age, gestational age, pregnancy times,  birth times | Sort of PA |  |  |  |  |  |  |  |
| ***[List outcome variables]*** |  |  | fetal radiation dose |  | 1, 5 and 10 minutes Apgar scores | balloon occlusion time | the occurren-ce of cesarean hysteric-tomy | estimated blood loss, Blood transfusion volume, Operating time | Length of stay in ICU,  Total hospital stay, vein thrombosis of lower limbs |
| ***[List other data variables]*** |  |  |  |  |  | Balloon occlusion-relative complications: vascular aneurysm, dissection or rupture, arterial embolism |  |  | Mortality, Menstruation and uterine follow-up |

*Recommended content can be displayed using various schematic formats. See SPIRIT 2013 Explanation and Elaboration for examples from protocols.

**List specific timepoints in this row.
